# Serial snapshot crystallography for materials science with SwissFEL

**DOI:** 10.1107/S2052252515006740

**Published:** 2015-04-21

**Authors:** Catherine Dejoie, Stef Smeets, Christian Baerlocher, Nobumichi Tamura, Philip Pattison, Rafael Abela, Lynne B. McCusker

**Affiliations:** a Laboratory of Crystallography, ETH Zurich, Vladimir-Prelog-Weg 5, Zurich, 8093, Switzerland; b Advanced Light Source, Lawrence Berkeley National Laboratory, 1 Cyclotron Road, Berkeley, CA 94720, USA; c Swiss-Norwegian Beamlines, European Synchrotron Radiation Facility, 71 avenue des Martyrs, Grenoble, 38042, France; d Laboratory of Crystallography, EPFL, Rte de la Sorge, Lausanne, 1015, Switzerland; e SwissFEL, Paul Scherrer Institut, Villigen PSI, 5232, Switzerland

**Keywords:** serial snapshot crystallography, multi-microcrystal diffraction, indexing, broad-bandpass beam, XFEL

## Abstract

New opportunities for studying (sub)microcrystalline materials with small unit cells, both organic and inorganic, will open up when the X-ray free electron laser (XFEL) presently being constructed in Switzerland (SwissFEL) comes online in 2017. Our synchrotron-based experiments mimicking the 4%-energy-bandpass mode of the SwissFEL beam show that it will be possible to record a diffraction pattern of up to 10 randomly oriented crystals in a single snapshot, to index the resulting reflections, and to extract their intensities reliably. The crystals are destroyed with each XFEL pulse, but by combining snapshots from several sets of crystals, a complete set of data can be assembled, and crystal structures of materials that are difficult to analyze otherwise will become accessible. Even with a single shot, at least a partial analysis of the crystal structure will be possible, and with 10–50 femtosecond pulses, this offers tantalizing possibilities for time-resolved studies.

## Abstract 2

We have mimicked the 4%-energy-bandpass mode of the SwissFEL beam at a synchrotron facility, collected snapshots of randomly oriented multi-crystal samples, and developed a methodology to index and process such non-monochromatic data for materials science applications.

## 1. Introduction   

Microcrystalline materials, with crystallite sizes in the micron to submicron range, are prevalent in many areas of science, including biology, chemistry, materials science and mineralogy. In each of these fields, structure elucidation is central to the understanding of a particular material’s properties. When single crystals large enough for conventional structure analysis are not available, powder diffraction techniques are usually applied, and indeed over the last two decades methods for data collection and structure determination have become increasingly powerful, allowing quite complex structures to be solved (David & Shankland, 2008 ▶). Nonetheless, powder diffraction techniques reach their limits in the case of complex structures with many atoms in the asymmetric unit and disordered materials. With the introduction of dedicated synchrotron radiation sources in the 1980s, the potential for monochromatic single-crystal microdiffraction was recognized and explored immediately (Eisenberger *et al.*, 1984 ▶; Rieck *et al.*, 1988 ▶; Andrews *et al.*, 1988 ▶; Harding, 1996 ▶). Nowadays, most synchrotron facilities have at least one beamline dedicated to monochromatic microcrystal diffraction. However, the size of the microcrystals used for most measurements is still larger than 500 µm^3^. This is because the handling and mounting of tiny crystals are delicate operations, the data collection requires that the crystal be rotated within the very small X-ray beam in a controlled manner, and the risk of radiation damage may increase as the crystal size decreases.

The white beam, Laue diffraction technique is an attractive alternative to the monochromatic one, because it takes full advantage of the X-ray energy spectrum at a synchrotron and requires no rotation of the crystal. With the white beam provided at a synchrotron source, a reasonably large amount of data can be collected in a single shot. The acquisition time is fast, and only a few patterns recorded on stationary randomly oriented crystallites are necessary to get a full dataset (Cornaby *et al.*, 2010 ▶; Dejoie, McCusker, Baerlocher, Kunz & Tamura, 2013 ▶). This makes the method attractive for the structural characterization of beam sensitive crystals (Cornaby *et al.*, 2010 ▶) and for *in situ* (Perry *et al.*, 2014 ▶) or time-resolved studies (Yorke *et al.*, 2014 ▶). One of the drawbacks of the method is the complexity of the data analysis, caused by the energy dependence of correction factors, and the overlap of harmonic reflections (Helliwell *et al.*, 1989 ▶). Furthermore, even though more data can be collected in a single shot without rotating the crystal, the duration of the exposure to a white synchrotron beam is sometimes still long enough for radiation damage to occur.

Another way of collecting microcrystal diffraction data is by using a suitably equipped electron microscope. This allows extremely small crystallites (~100 nm on an edge) to be examined, because electrons interact with matter much more strongly than do X-rays. Unfortunately, precisely because of this strong interaction, the electron diffraction intensities can be severely distorted by the effects of dynamical and multiple scattering, and this complicates their use in structure analysis. This problem has been reduced with the introduction of the precession electron diffraction technique (Vincent & Midgley, 1994 ▶; Midgley & Eggeman, 2015 ▶), and three-dimensional electron diffraction datasets can now be collected, using either the method of automated diffraction tomography (Kolb *et al.*, 2007 ▶) or that of rotation electron diffraction (Zhang *et al.*, 2010 ▶). Over the last few years, an impressive array of structures from nanometer-sized crystals has been investigated successfully (Gorelik *et al.*, 2012 ▶; Willhammar *et al.*, 2014 ▶). However, these methods are not applicable to all materials, such as beam sensitive materials or materials requiring a controlled atmosphere. The non-kinematical intensities also remain an issue, because the intensities are not reliable enough to allow the structure to be fully refined, although progress has also been made in this area (Palatinus *et al.*, 2015 ▶).

With the development of X-ray free-electron laser (XFEL) sources, which create ultra-fast X-ray pulses of unprecedented brilliance, a new option for the structural characterization of microcrystalline materials is arising. By exposing a small crystallite (from nm to a few µm) to a single monochromatic pulse of tens of femtoseconds in duration, a diffraction pattern can be obtained before the crystal is damaged. If such single-pulse diffraction patterns, collected sequentially on many randomly oriented crystallites, are combined, it is possible to determine the crystal structure (Chapman *et al.*, 2011 ▶; Boutet *et al.*, 2012 ▶). In a typical serial femtosecond crystallography (SFC) experiment, hundreds of thousands of individual snapshots are collected from a flowing suspension of nanocrystals in a liquid jet. Several analytical tools and dedicated algorithms have been developed to filter the hundreds of thousands of frames collected, to index individual snapshots and to reconstruct the reflection intensities using a Monte Carlo integration method (White *et al.*, 2012 ▶, 2013 ▶; Hattne *et al.*, 2014 ▶; Brewster *et al.*, 2015 ▶). The latter is crucial for further use of the data, because only a single slice of the Ewald sphere is accessed in each diffraction pattern, so the reflections are only partially measured.

To date most activity in the realm of structure analysis using XFEL radiation has focused on monochromatic beams and macromolecular systems (Barends *et al*., 2014 ▶). Our prime interest is in the area of organic and inorganic materials, where the diffraction patterns are much sparser. One way of increasing the number of reflections in diffracting condition is to use a larger energy bandpass, as in a Laue diffraction experiment. This capability will be available to a limited extent at the SwissFEL facility (PSI, Switzerland), a new XFEL source scheduled to come online in 2017 (Patterson *et al.*, 2010 ▶; Patterson *et al.*, 2014 ▶). Indeed, one of the unique features of the SwissFEL beam will be that the bandpass of the X-ray beam can be adjusted to give as much as a 4% energy spread.

With the 4%-energy-bandpass mode, not only can more reflections be recorded per shot, but the intensities will also be measured more reliably (Dejoie, McCusker, Baerlocher, Abela *et al.*, 2013 ▶; White *et al.*, 2013 ▶). The broader range of energies puts more reflections in diffracting condition and simultaneously allows the full width of most reflections to be measured, making integration easier. This provokes the question as to whether or not the SFC approach using a broad bandpass beam could be applied to materials with smaller unit cells (up to *ca* 25000 Å^3^). Computer simulations indicate that with such a setup, a full dataset can be obtained with just a few hundred crystals, instead of tens or hundreds of thousands (Dejoie, McCusker, Baerlocher, Abela *et al.*, 2013 ▶).

Here we describe the application of this approach to experimental data collected on three inorganic materials at a synchrotron source under conditions mimicking the SwissFEL beam. We have developed new algorithms to index the experimentally measured single-shot patterns of randomly oriented crystals of such materials, and show that the measured intensities are accurate and suitable for structure analysis. To improve the efficiency of the serial snapshot crystallography data collection for materials with small unit cells, we also investigated the possibility of collecting and analyzing data from more than one randomly oriented crystal in a single snapshot.

## 2. Mimicking the SwissFEL beam on SNBL at ESRF   

**Figure 1:**
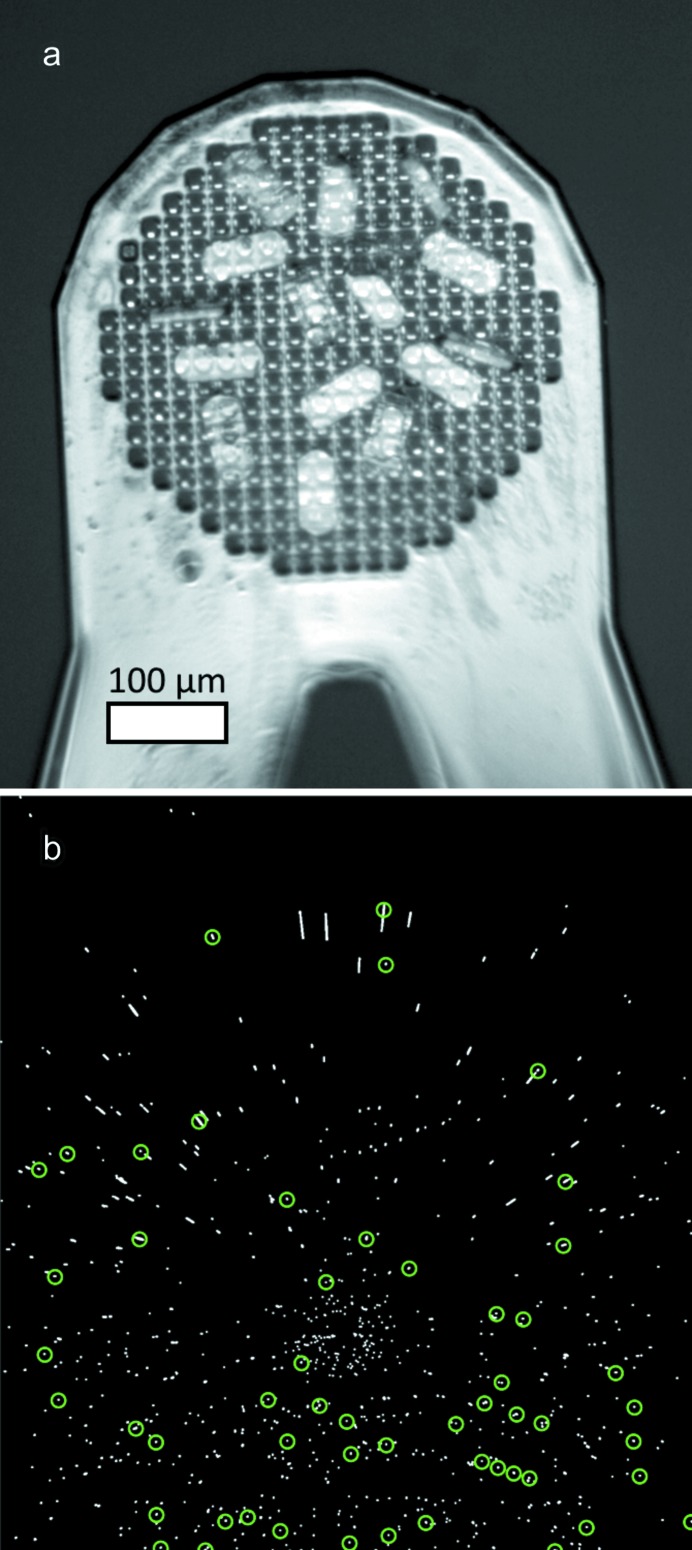
(*a*) 15 crystals of ZSM-5 dispersed on an MiTeGen grid. (*b*) Typical frame showing data collected on 15 crystals of ZSM-5. There are a total of 664 observed reflections, and 58 that originate from one of the crystals, are indicated with green circles.

At present, there is no experimental facility that can produce an intense X-ray beam with a 4% energy bandpass directly, so the single-crystal diffractometer on the Swiss–Norwegian Beamlines (SNBL/BM01A) at the European Synchrotron Radiation Facility (ESRF) was used to mimic the SwissFEL setup experimentally. The broad bandpass mode was achieved by collecting a diffraction pattern while the monochromator was scanned over a 4% energy range (average energy 17.34 keV, or 0.7153 Å). Because the SNBL setup was not suited for snapshot data collection on a series of individual crystals, the 4% bandpass patterns were collected by rotating single crystals mounted on MiTeGen MicroMeshes (Fig. 1 ▶). A first series was obtained by taking a ‘snapshot’ after each 1° rotation around the *ϕ* axis of the goniometer (*i.e.* 360 different frames or crystal orientations). In order to simulate the case of crystals with platelike morphology which would probably display a preferential orientation on a flat surface, a second series of measurements was carried out by rotating around a single face of one of the crystals (*ψ* scan). A conventional monochromatic *ϕ* scan (0.25° steps) was also performed on the same test crystals to obtain reference datasets. A two-dimensional Dectris Pilatus 2M detector was positioned at a distance of 144 mm from the sample for the *ϕ* scans, and at 224 mm for the *ψ* scans. Geometry calibration (sample-detector distance, normal incidence position of the detector, tilt angle of the detector) was carried out with the *XMAS* program (Tamura, 2014 ▶), using an LaB_6_ reference powder pattern.

Three test samples were used: the zeolite ZSM-5 (Olson *et al.*, 1981 ▶), a hydrated caesium cyanoplatinate (Cs_2_[Pt(CN)_4_]·H_2_O) (Johnson *et al.*, 1977 ▶), and the mineral sanidine (Ackermann *et al.*, 2004 ▶). As with all cyanoplatinates, Cs_2_[Pt(CN)_4_]·H_2_O is toxic if swallowed. Appropriate laboratory safety procedures apply when handling this family of compounds. These materials have unit cells typical of small-molecule and inorganic structures and cover three different crystal systems (Table 1 ▶). To compensate for the much lower flux of the bending-magnet synchrotron beam (at least four orders of magnitude lower than that expected for SwissFEL), relatively large single crystals (*ca* 15000 µm^3^) were used. In order to test the possibility of indexing multiple patterns, two grids with multiple randomly dispersed crystals were also prepared, one with three ZSM-5 crystals on a grid and one with 15 (Fig. 1 ▶).

## 3. Indexing single snapshot patterns   

**Figure 2:**
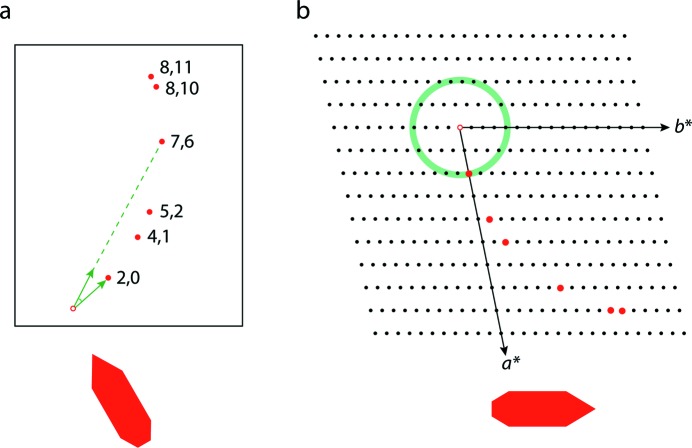
Schematic drawing of the indexing procedure for 4%-energy-bandpass data in two dimensions. (*a*) Single snapshot (top) for a randomly oriented crystal (bottom), and (*b*) its interpretation in reciprocal space (top) with the reference orientation of the crystal (bottom). The origin in both cases is shown as an open red circle. In (*a*) two **q** vectors and the angle between them are highlighted in green (used in the *laue* approach). In (*b*) a green circle with the *d* spacing of the reflection closest to the origin in (*a*) is shown. All reciprocal lattice points on this circle are considered to be potential candidates for that reflection (used in the *mono1* approach). The indices of the reflections are given in (*a*) and their positions in the reference orientation are shown in (*b*).

Indexing the single snapshots involved overcoming four problems: (1) an accurate orientation had to be retrieved from a single frame, (2) the data were collected using a 4% energy bandpass, so the wavelength associated with each diffraction spot is indeterminate, (3) organic/inorganic materials typically have small unit cells, so the number of observations per frame is limited, and (4) the orientations of multiple crystals should be determined from a single frame. It is normally possible to obtain unit-cell parameters from a powder diffraction pattern, so this information was used as *a priori* knowledge to index the 4% bandpass data. Two ways of dealing with the data were investigated. One approaches the problem from a polychromatic point of view using a modification of an algorithm developed at the Advanced Light Source to index Laue microdiffraction data (Tamura, 2014 ▶), and the other from a more conventional monochromatic point of view, assuming an average wavelength (Fig. 2 ▶).

### 3.1. Laue approach   

Our first intuition was to use the tools developed by the Laue microdiffraction community, where finding orientations of crystals with known cell parameters from a single frame is common practice (Chung & Ice, 1999 ▶; Tamura, 2014 ▶). Typically, the indexing procedure consists of matching the experimental pattern with the corresponding portion of the reciprocal lattice calculated from the unit-cell parameters. Since the exact energy associated with each reflection is not known, only the directions of the reciprocal lattice vectors can be used. The angles between these vectors, which are unaffected by the energy of the incident radiation, are then compared with a calculated list of angles generated from the known unit cell. This approach, implemented in the *XMAS* software, was developed for indexing Laue patterns (5–24 keV range) of small-unit-cell samples of known structure (*i.e.* reflection intensities can be calculated). These assumptions made the indexing of 4% bandpass data of samples of unknown structure slow and highly unreliable.

To overcome the problems, some new features were introduced into the conventional angle computation method. Instead of classifying the reciprocal lattice points generated from the unit-cell parameters according to their associated structure factors, the points are now sorted by decreasing *d* spacing, so no *a priori* knowledge of the structure is required. The second major modification is the introduction of a three-dimensional pattern-matching algorithm to recover the orientation matrix.

The indexing process is started by selecting a set of measured reflections with the highest intensities and a set of calculated reciprocal lattice points with the largest *d* spacings. The number depends on the sample, the quality of the data, and the number of crystals measured. The selected reflections are converted into normalized reciprocal space vectors **q**_norm_ (*i.e.* the magnitudes are set to 1) and the reciprocal space points to vectors **q**. A three-dimensional pattern-matching algorithm, adapted from the one described by Van Wamelen *et al.* (2004 ▶) in two-dimensional space, is then applied. First, a pair of **q**_norm_ vectors defining an angle is selected from the measured data (starting from the strongest reflections), and the observed vectors in the immediate vicinities of these two are identified. The ‘distance criteria’ defining the local environment of the selected pair of **q**_norm_ vectors has to be chosen carefully to keep the computation time low without decreasing the indexing success rate. This ‘first neighborhood’ is then compared with calculated vector pair environments to find local matches. These local matches are classified according to their goodness-of-fit, and then the global match including all vectors is examined. The algorithm stops when a sufficiently good global match (number of indexed peaks) is found. The orientation matrix obtained is refined, a check for reflections not indexed in the first step is performed, and then the orientation matrix is refined again. If the parameters are properly defined (in particular the number of reflections used and the distance criteria), this nearest-neighbors approach is considerably more efficient and faster than the conventional alignment-type approach used in most Laue indexing algorithms.

In the case of multiple crystals per frame, a sequential approach is used. As soon as the first set is identified, the indexed peaks are removed from the original list of measured reflections, and the indexing process is repeated.

This Laue-based indexing algorithm is implemented in Fortran 90, as part of the *XMAS* software package.

### 3.2. Monochromatic approach   

The 4% bandpass data are quite close to monochromatic, and can be interpreted as such by assuming an average wavelength for each reflection. We examined several methods used to index conventional monochromatic data, some of which are also being used through the *CrystFEL* or the *cctbx.xfel* programs to index single frames collected on macromolecular materials in XFEL experiments (White *et al.*, 2012 ▶; Hattne *et al.*, 2014 ▶). These indexing methods typically convert a set of positions of recorded reflections into reciprocal-space vectors and analyze them for periodicity in order to determine the basis vectors and to find the metric and orientation of the unit cell. In *XDS* (Kabsch, 1988 ▶, 1993 ▶, 2010 ▶), differences between reciprocal-lattice vectors are calculated and accumulated in a histogram. The resulting clusters allow a unique basis to be determined. In the case of 4% bandpass data, this method was quickly abandoned, because it yielded streaks of reflections, rather than clusters – an effect of averaging the wavelength. Another approach was developed by Duisenberg (1992 ▶) in the *DIRAX* program, where row periodicity is sought by projecting all observed reciprocal lattice vectors onto the normal to the plane given by three randomly selected points. Later methods implemented in *MOSFLM* (Steller *et al.*, 1997 ▶; Leslie, 2006 ▶), *DENZO* (Otwinoswki & Minor, 1996 ▶; Otwinoswki *et al.*, 2012 ▶) and *LABELIT* (Sauter *et al.*, 2004 ▶) followed a similar approach, but probed all possible directions and used a fast Fourier transform (FFT) to look for periodicity. These methods gave some useful results for 4% bandpass data for frames obtained from a single-crystal, but the majority of the frames simply did not contain enough reflections to determine at least two independent base vectors, and there was no guarantee that the method would scale well to multiple crystals.

It became clear that these monochromatic indexing methods were ill-suited for indexing single snapshots of organic/inorganic materials taken with broad bandpass radiation. Therefore, a strategy to deal specifically with such data was devised. The indexing procedure was split into two parts. The first part entails a search for suitable orientation matrices, and the second the evaluation of each orientation matrix.

For the first part, two different approaches were developed (designated *mono1* and *mono2* for convenience). The *mono1* algorithm resembles a classic approach reported by Busing & Levy (1967 ▶) and Sparks (1976 ▶) some 40 years ago, in which the crystal orientation is determined by assigning indices to two low-resolution non-collinear reflections manually. In our case, the initial indices are not known, but the lattice spacings for all reflections are. Therefore, each reflection can be assigned a set of pre-calculated indices (those with similar *d* spacings). A semi-exhaustive search for the orientation matrix is then performed by trying all combinations of non-collinear spots and their corresponding indices. The angle between the **q** vectors is independent of the incident radiation, so only pairs of reflections where the angle of the observed reflections closely matches those of their tentative indices are kept. For each pair, the orientation of the crystal can then be determined. Although the algorithm is fast and reliable for one crystal per frame, it was found to scale poorly with increasing number of diffracting crystals. Therefore, a second routine, *mono2*, was developed. This is a ‘brute-force’ approach that simply samples a fine grid of all possible crystal orientations. Steller *et al.* (1997 ▶) described an algorithm for generating all possible directions on a hemisphere. With this algorithm and a spacing of approximately 1.7° (0.03 radian) between vectors, a sufficiently dense coverage is achieved, and by adding a stepwise rotation (again *ca* 1.7° increments) of 360° around each of these vectors, roughly 1.5 million equally distributed rotation matrices that cover all possible crystal orientations can be generated efficiently.

Both the *mono1* and *mono2* approaches yield a large number of candidate orientation matrices, and these are evaluated in the second part of the routine. First, each candidate orientation matrix is used to transform the **q** vectors of the measured reflections to indices, and this will normally yield non-integer values. A reflection is considered indexed if all three indices *hkl* are close to integer values. *nfit* is the number of indexed reflections in the pattern, and *score* gives the residual sum of the squared differences of the distances between the observed and calculated indices. To keep computation times low, each orientation matrix with *nfit* below a certain threshold value *nmin1* is discarded. For each remaining solution, the rotation matrix is optimized against *score*/*nfit* ^2^ using a least-squares routine. This refinement step significantly improves the number of indexed peaks, despite the fact that the average (monochromatic) wavelength is still used. At this point, the wavelength for each reflection is recovered by minimizing the distance between the indices of a reflection and its nearest integer equivalent. The *score* and *nfit* values for each optimized orientation matrix are recalculated, and again, orientation matrices with *nfit* below a second threshold value *nmin2* (typically higher than *nmin1*) are discarded. Equivalent solutions are merged according to the Laue symmetry of the space group.

All remaining independent solutions are then ranked by *score*/*nfit* ^2^. For one crystal in the beam, simply the highest ranked orientation matrix is taken. To handle measurements of multiple crystals, a greedy weighted set-optimization algorithm (Young, 2008 ▶) was implemented to identify the smallest subset of orientation matrices that indexes the largest number of reflections. This last algorithm had to be modified to take into account that not all crystal orientations are necessarily found, so a number of reflections may be left unindexed.

The main difficulty in the monochromatic approach is to define meaningful values for the thresholds *nmin1* and *nmin2*, especially when dealing with a large number of frames with a fluctuating number of crystals, incident flux, and scattering power of the crystals. In order to estimate an appropriate starting value, the brute force algorithm can be run with large angle increments (17°, 0.3 rad) to probe a representative distribution of approximately 1500 orientations. The *mean* and standard deviation (*sd*) of all corresponding values of *nfit* are calculated. In this way, the approximate number of reflections that can be indexed with incorrect orientations can be estimated for a particular frame (*i.e.* its ‘noise’ level). A meaningful formula for *nmin1* was found empirically to be *mean* + 3 × *sd*. By applying this formula, the indexing algorithm can adjust to arbitrary variations in the data automatically, and this improves the effectiveness of the routine considerably.

The *mono1* and *mono2* algorithms are implemented in Python2.7, using the pandas, numpy and scipy libraries. Crystallographic computations are performed using the *cctbx* software (Grosse-Kunstleve *et al.*, 2002 ▶).

## 4. Application to test data   

### 4.1. Indexing results   

The three algorithms were evaluated using data collected on three test samples. Indexing results are reported in Table 2 ▶. For ZSM-5, with the largest unit cell, all algorithms work well, but the monochromatic approaches are faster. The *a* and *b* axes of ZSM-5 differ by only 0.5%, but all three algorithms could assign the axes correctly in almost all cases, despite the fact that there is a 4% range of energies. The unit cell of Cs_2_[Pt(CN)_4_]·H_2_O is much smaller, so the number of reflections per frame decreased. This made very little difference to the *laue* approach, except to make it faster, but did affect the *mono1* algorithm, where only 205 of the 360 frames could be indexed. Of these, however, 99% were correct. For sanidine, with the smallest unit cell, only 29% of the frames could be indexed using the *mono1* method, but all of these were indexed correctly. The *mono2* algorithm, however, had no problem dealing with the smaller unit cells, and correctly indexed all frames for both materials. With the *laue* approach, 79% of the sanidine frames could be indexed correctly in a short period of time, but the increasing number of false positive results may be an indication that the indexing procedure approaches its limits with such small unit cells.

The three algorithms were also applied to multi-crystal data (Table 2 ▶, Fig. 1 ▶) collected on the ZSM-5 sample. Both monochromatic approaches could index the multicrystal frames successfully, but the *mono2* algorithm worked considerably better and was easier to set up. With the *mono2* algorithm, it was possible to retrieve a large number of crystal orientations while maintaining reasonable computation times with a desktop computer. With three crystals on the grid, on average 2.8 crystal orientations per frame could be recovered. With 15 crystals, 8.5 crystal orientations per frame could be retrieved for 12 independent crystals. Although 15 crystals can be seen on the grid (Fig. 1 ▶ *a*), 3 of them do not diffract strongly enough. It was also possible to use the *laue* approach for multiple crystals, but the computation time became a limiting factor, so the tests with 15 crystals were not pursued. As before, the main source of error was the similarity between the *a* and *b* parameters. It was noted, however, that as the number of crystals increased, so did the potential for incorrect assignment of indices, because the chances of reflections from different crystals accidentally overlapping increased. We estimate that with the present setup between 5 and 10 crystals per shot would be optimal, but by using larger detectors with higher resolution, this number will certainly increase. It is perhaps important to note that fine tuning is possible in all algorithms, but we were interested in having a fully automated robust procedure that would be able to process a large amount of data without intervention. The main point is that reflections on most of the single snapshot images can indeed be indexed, despite the differences in unit cell, symmetry and number of crystals, and this means that a useful dataset can be extracted reliably for different materials.

We also tested the monochromatic based algorithms on pure monochromatic data (*ϕ* scan) collected on all 3 samples. Although the algorithms were optimized to deal with a 4% bandpass, they still performed satisfactorily in indexing these data. We therefore reason that single frames collected with any bandpass between 0 and 5% can be indexed successfully. For energy bandpass higher than 5%, we expect a Laue approach to be better.

### 4.2. Limitations   

As mentioned earlier, increasing the number of crystals diffracting simultaneously increases the probability of accidental overlap of reflections from different crystals, and this will affect both the indexing process and the intensity extraction. In particular, in the case of the monochromatic indexing approach, the least-squares refinement of the rotation matrix may be affected if alien peaks are close to the reciprocal lattice points of the crystal being refined. This effect is exacerbated with plate-like crystals dispersed on a grid, where most will lie on the same face and then only be randomly oriented around the normal of the face. This is the case for the two multi-crystal ZSM-5 preparations (Fig. 1 ▶), for which 2 of the 3 crystals and at least 9 of the 15 crystals share a similar orientation of the *b* axis.

To investigate the effect of flat or needle-shaped crystals dispersed on a grid, 4% bandpass data were collected on a single-crystal of ZSM-5 in a *ψ*-scan mode (rotation around one face of the crystal), and results were compared with data collected with a more conventional *ϕ* scan. The geometry of the experimental setup (*ψ*-scan mode) only allowed a 90° rotation. Nonetheless, the effect on the completeness of the data (number of unique reflections measured *versus* total number expected), calculated in the 1.0–5.0 Å resolution range (where it is maximal), was not as dramatic as might be expected. The values of 54% and 60% were obtained for the *ψ*-scan and *ϕ*-scan datasets, respectively. For a real SFC experiment, of course, a sample mount that reduces preferred orientation of the crystals should be used.

Because a broad bandpass beam was used, specific problems inherent to Laue diffraction have to be checked. One of them is the possible presence of harmonics (*e.g.* the reflections 100, 200, and 300 will have identical diffraction angles for wavelengths *λ, λ*/2, and *λ*/3, respectively). The formula 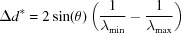 gives the relationship between the minimum distance between two reciprocal lattice points Δd*, the diffraction angle *θ*, and the wavelengths of the two delimiting Ewald spheres *λ*_min_ and *λ*_max_. The maximum diffraction angle *θ* that can be used to suppress all harmonics can then be calculated for any given unit cell and wavelength range. For ZSM-5, the minimum distance between two reciprocal lattice points is given by |a*|, where *a* = 20.0022 Å (Table 1 ▶). This gives a maximum 2*θ* angle of 46.2° for *λ*_average_ = 0.7153 Å (*d* spacing = 0.9116 Å). With the current setup (sample–detector distance 144 mm), 93% of all reflections fall below this threshold, and only the *h*00 and 0*k*0 reflection classes are affected at the higher angles. When SwissFEL comes online, the minimum wavelength will be close to 1.000 Å, so the probability of recording harmonic reflections will be reduced even further.

### 4.3. Structure solution and refinement   

To test the data analysis procedures beyond the indexing step and to evaluate the quality of the measured data, reflection intensities for the indexed patterns were integrated using the seed-skewness method (Bolotovsky *et al.*, 1995 ▶). The patterns indexed with the *laue* approach were used for this purpose. Reflection intensities were corrected for various factors using techniques similar to those used for Laue microdiffraction data (Dejoie *et al.*, 2011 ▶). In particular, each frame was corrected for the decay of the intensity of the incident flux with time, and for the variation of the incident flux with energy. All frames with less than 20 indexed reflections for ZSM-5 and 6 indexed reflections for sanidine and Cs_2_[Pt(CN)_4_]·H_2_O were discarded. Six incorrectly indexed patterns remained in the final dataset for ZSM-5, as a result of the *a* and *b* axes being reversed. On the other hand, all frames for sanidine and Cs_2_[Pt(CN)_4_]·H_2_O in the final dataset were correctly indexed. As the volume of the crystal was constant in this experiment, no scaling between the individual frames was applied. The situation will be different at SwissFEL, where each crystal can have a different volume and where the variation of the incident flux from shot to shot will be significant. These problems will be addressed in a future publication.

**Figure 3:**
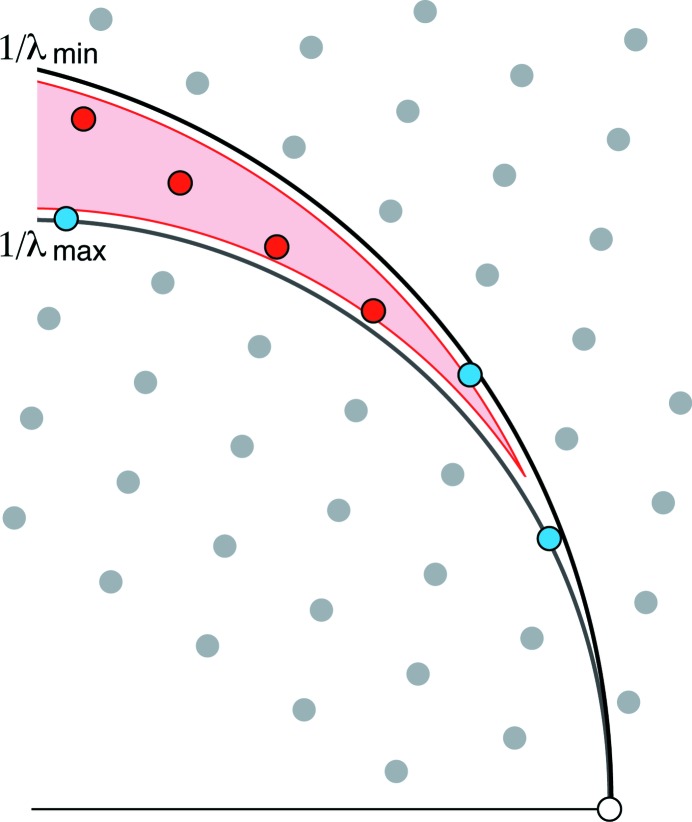
Ewald construction showing the 4%-energy-bandpass experiment (proportions exaggerated to show more detail). The reciprocal lattice points are shown as large dots to indicate that the intensities associated with them have a finite width. For simplicity, the reflections here are assumed to be isotropic and the widths identical for all reflections. The white borders between the Ewald spheres delimited by 1/*λ*_min_ and 1/*λ*_max_ and the shaded area correspond to half the reflection width (see main text). Here, four reflections are fully measured (red) and three partially (blue).

In order to identify the fully measured reflections in each dataset, the wavelength associated with each reflection (determined during the indexing process) was examined. Those lying close to the wavelength boundaries are most likely to be partially measured (Dejoie, McCusker, Baerlocher, Abela *et al.*, 2013 ▶). A simple schematic drawing is shown in Fig. 3 ▶, where the width of the white border between the shaded region and the delimiting Ewald spheres corresponds to the radius of a reflection. If the center of a reflection is within the shaded area it will be fully measured. Of course, not only do reflection widths vary from sample to sample, they can also vary with *hkl* and they may be asymmetric, so the simple picture given in Fig. 3 ▶ may need to be modified for real data. Note that reflections close to the origin of the reciprocal lattice will never be fully measured in a single shot. On the other hand, an increasing number of reflections are fully measured as the diffraction angle increases.

**Figure 4:**
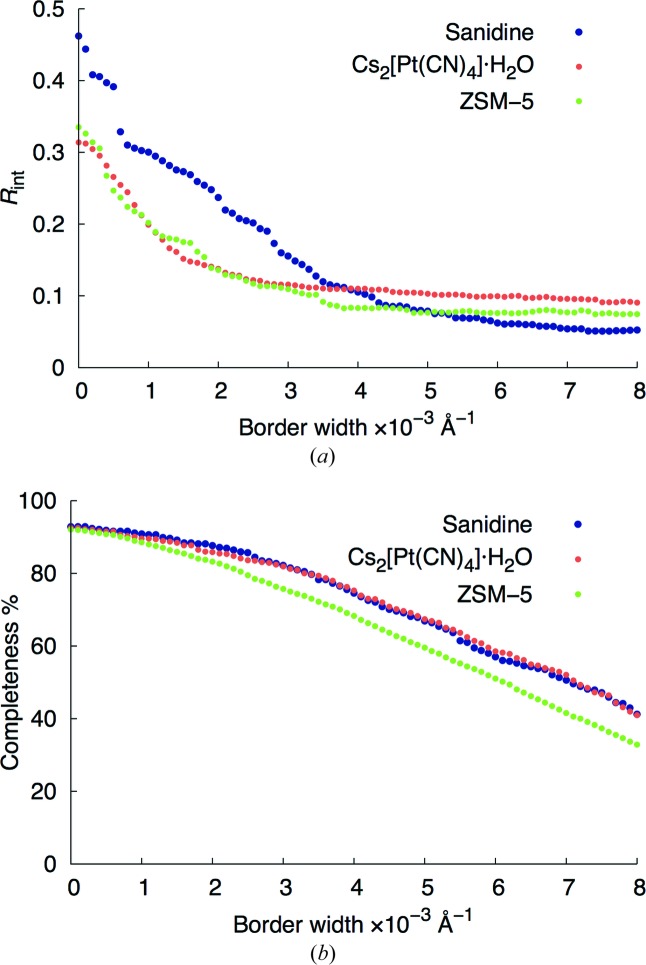
Changes in (*a*) *R*_int_ and (*b*) completeness as a function of the size of the border (see Fig. 3 ▶). Both are calculated up to a resolution of 1 Å.

Datasets with increasingly larger borders were constructed. The internal *R* value (*R*_int_) and completeness provided by the *cctbx* software (Grosse-Kunstleve *et al.*, 2002 ▶) were used to monitor the datasets up to a resolution of 1.0 Å (Fig. 4 ▶). For all samples, a clear decrease in *R*_int_ is observed. An *R*_int_ of ~10% (or less) is achieved for all three samples with a border of 2×10^−3^ Å^−1^. This level is directly related to the elimination of partially measured reflections near the two boundaries of the energy range and close to the origin of the reciprocal lattice. The corresponding completeness values are still more than 80% for these reduced datasets (Fig. 4 ▶ *b*). On the other hand, the resolution range gets smaller, because the lower resolution reflections are only partially measured and are therefore removed. As a result, the lower resolution limits for sanidine, Cs_2_[Pt(CN)_4_]·H_2_O, and ZSM-5 decrease from 6.648 to 2.234 Å, 7.777 to 2.344 Å, and 11.126 to 2.653 Å, respectively.

**Figure 5:**
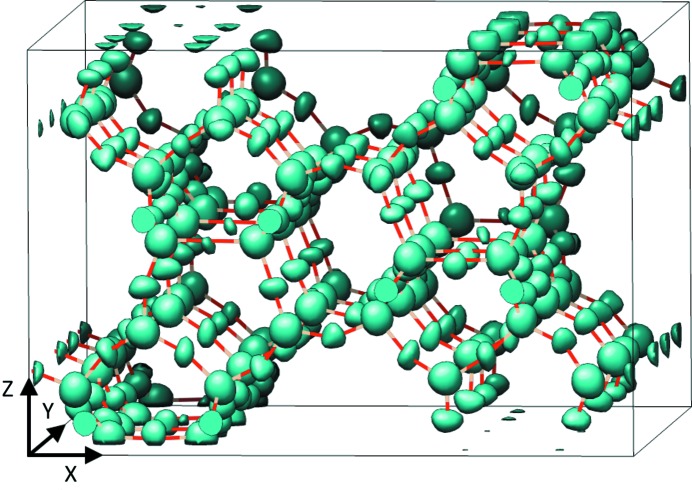
Electron density map generated by the charge-flipping algorithm in *Superflip* using 4%-energy-bandpass data (full dataset) collected on the zeolite ZSM-5. The refined structure has been overlaid for comparison.

Full datasets (partially measured reflections included) and reduced datasets were used for structure solution using the charge-flipping algorithm (Oszlányi & Sütő, 2004 ▶) in *Superflip* (Palatinus & Chapuis, 2007 ▶). The structures of the three samples could be solved in all cases, despite the presence of partially measured reflections in the full datasets. The electron density map obtained with the ZSM-5 data is shown in Fig. 5 ▶ to give a visual impression of the quality of the data.

**Figure 6:**
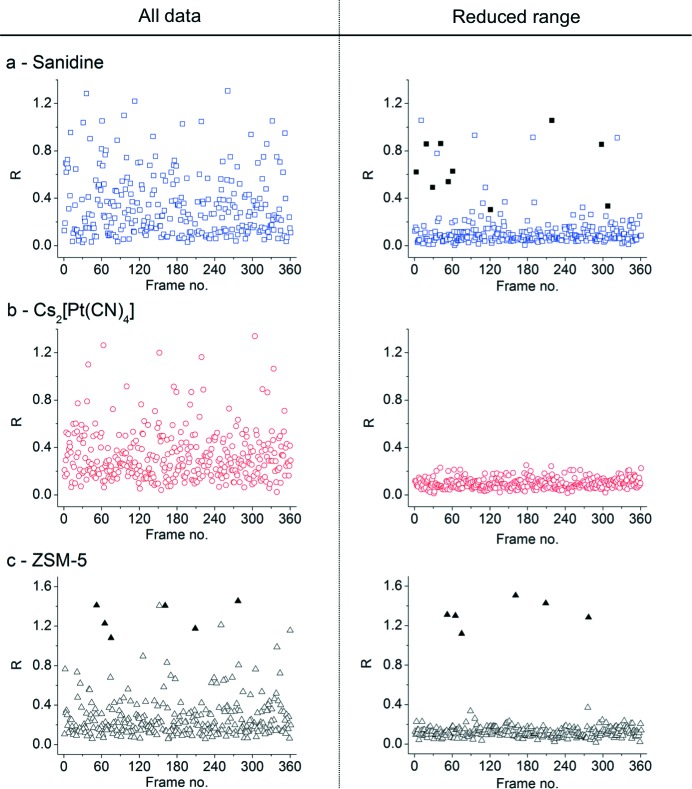
*R* values for the 360 individual frames for (*a*) sanidine, (*b*) Cs_2_[Pt(CN)_4_]·H_2_O, and (c) ZSM-5. In each case, the results for all data are shown on the left and for the reduced dataset (2×10^−3^ Å^−1^ border for ZSM-5, and 2.5×10^−3^ Å^−1^ for sanidine and Cs_2_[Pt(CN)_4_]·H_2_O) on the right. For sanidine, the outliers that are removed by increasing the border further are shown as filled squares. For ZSM-5, the incorrectly indexed patterns (*a* and *b* reversed) are indicated with filled triangles.

After structure solution, an agreement value
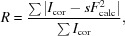
where 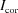 is the corrected integrated intensity, *s* is the scale factor and 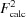 is calculated from the structural model (obtained in the structure solution step), was calculated for each frame, in order to identify any outlying frames (*i.e.* frames with one or more partially measured reflections). Individual agreement values calculated for the full dataset and for a reduced dataset are shown graphically for all three samples in Fig. 6 ▶. For each sample, the optimal reduced dataset was chosen as a compromise between completeness (≥ 80%) and quality (*R*_int_ ≤ 10%). With the full datasets, the agreement values are highly dispersed (*R* up to 50%), and many outliers (*R* > 50%) are present. However, with the reduced datasets, most of the agreement values lie below 25%, and only a few outliers (*R* > 30%) can be seen. This is consistent with the presence of partially measured reflections at the borders of the energy range and close to the origin of the reciprocal lattice in the full datasets. For ZSM-5, the six frames in which *a* and *b* were wrongly assigned in the indexing process are the most obvious outliers. The other two with agreement values slightly above 30% result from the presence of a single partially measured reflection still remaining in each frame. For Cs_2_[Pt(CN)_4_]·H_2_O, no outliers can be spotted in the reduced dataset. For sanidine, the number of outliers in the reduced dataset is larger than for the other two samples. This is related to the presence of one or two partially measured reflections in these particular frames. The reason for the partial measurement of these reflections is mainly related to the intrinsic breadths of the sanidine reflections. From the conventional monochromatic datasets, the average widths of the Bragg reflections were extracted for each of the three test samples using the *CrysAlis* software (Oxford Diffraction, 2008 ▶) (Table 3 ▶). The widths of the sanidine reflections are clearly larger than those of ZSM-5 and Cs_2_[Pt(CN)_4_]·H_2_O, so for sanidine the border at the boundaries needs to be increased even further, if only fully measured reflections are to be included. This can also be seen in Fig. 4 ▶, where the *R*_int_ value reaches a plateau when all reflections on all frames within a dataset are fully measured. This happens at a much larger border size for sanidine than for the other two samples.

Structure refinements were then performed with the *SHELX* software (Sheldrick, 2008 ▶) using the reduced datasets (2×10^−3^ Å^−1^ border for ZSM-5, and 2.5×10^−3^ Å^−1^ for sanidine and Cs_2_[Pt(CN)_4_]·H_2_O). Frames with an *R* value above 0.3 for ZSM-5 and sanidine were discarded. Atomic coordinates, displacement parameters, and an overall scale factor were allowed to vary. In all three structures, anisotropic displacement parameters were used for the cations and isotropic ones for the oxygen atoms. Refinement results are given in Table 4 ▶. Agreement values obtained from a conventional monochromatic refinement are also given for comparison. The *R* factors obtained after refinement with the reduced datasets are still slightly higher than those obtained with the monochromatic data. The main reasons for these differences are probably the presence of a few remaining partially measured reflections, the fact that the correction factors used after the extraction of the intensities still require further improvement, and/or the differences between the intensity extraction procedure used in *CrysAlis* and in our method.

## 5. Implications for the SwissFEL experiment   

In view of these results, an appropriate setup for a SwissFEL experiment for micron- or even submicron-sized crystals can be devised. Unlike proteins, most small-unit-cell materials are stable without a mother liquor, so they can be mounted on a grid similar to those used in an electron microscopy or a Laue microdiffraction experiment (Dejoie, McCusker, Baerlocher, Kunz & Tamura, 2013 ▶), or on some other low-background solid support (Ring *et al.*, 2011 ▶). For crystals with a needle- or plate-like morphology, which tend to orient preferentially, a support with a roughened surface can be used to obtain more orientations and/or the sample can be tilted with respect to the beam from frame to frame to increase the number of different orientations and thereby access a larger portion of reciprocal space. No special precautions need to be taken to ensure that only one crystal is irradiated per shot. On the contrary, it is actually an advantage to have more than one crystal in the beam, and our indexing algorithms can successfully deal with such data. Thus, simply by spreading the microcrystals on the sample support and translating the support between shots, useable data will be recorded on every frame, saving both beamtime and sample.

## 6. Conclusion   

The fact that single snapshot diffraction patterns from materials with small unit cells can indeed be indexed opens up new horizons for the structure analysis of polycrystalline materials. It means that high-quality single-crystal X-ray diffraction data can be obtained from tiny crystals in a very short period of time, and this has implications for all areas of materials research, particularly for beam-sensitive samples. Furthermore, because several crystals can be measured simultaneously, agglomerates do not pose a problem and a useful subset of a full dataset can be measured with a single 10–50 femtosecond pulse. The latter offers exciting possibilities in the area of time-resolved experiments. The unique 4%-energy-bandpass mode that is planned for the SwissFEL facility is particularly well suited for this kind of experiment, because it allows more reflections to be measured in a single shot, and they are measured completely. While our algorithms were developed with SwissFEL in mind, they can be applied to any data collected in single snapshot mode with a broad bandpass beam. We expect future studies using this technique to demonstrate just how diverse its application can be.

The indexing programs will be made available in the public domain, once they have been properly tested.

## Figures and Tables

**Table 1:** Unit cells of the three test samples

	Space group	*a* ()	*b* ()	*c* ()	** ()	Volume (^3^)
Sanidine	*C*2*/m*	8.5832	13.0076	7.1943	116.023	722
Cs_2_[Pt(CN)_4_]H_2_O	*P*6_5_	9.7910		19.5100		1620
ZSM-5	*Pnma*	20.0022	19.8990	13.3830		5327

**Table 2:** Results of the indexing tests (360 frames per data collection)

Sample	Average No. of reflections per crystal	Method	No. of patterns indexed	No. of correct solutions	Time† (per frame)
Sanidine	11	*laue*	332	285‡	1min
		*mono1*	105	105	4 s
		*mono2*	359	359	40 s
					
Cs_2_[Pt(CN)_4_]H_2_O	15	*laue*	360	357‡	1min
		*mono1*	205	203	5 s
		*mono2*	360	360	12 s
					
ZSM-5§ 1 crystal	44	*laue*	360	347	110min
		*mono1*	353	343	0.5 s
		*mono2*	360	351	12 s
					
ZSM-5§ 3 crystals	23	*laue*	835	791	1030min
		*mono1*	964	826	22 s
		*mono2*	1004	902	12 s
					
ZSM-5§ 15 crystals	57	*mono1*	2190	1965	55 s
		*mono2*	3027	2854	30 s

† Indexing trials were performed on a single core of a 2010 Mac Pro equipped with a dual 2.4GHz Quad-Core Intel Xeon processor.

‡ Solutions with less than 6 indexed peaks were discarded.

§ Incorrect solutions for ZSM-5 correspond to *a*/*b* flipping.

**Table 3:** Average reflection profile () extracted from monochromatic data Profiles were extracted using the program *CrysAlis* (Oxford Diffraction, 2008 ▶) (e1 and e2 are in the plane of the detector, and e3 is the scanning direction).

Direction	Sanidine	Cs_2_[Pt(CN)_4_]H_2_O	ZSM-5
e1	0.135	0.078	0.095
e2	0.166	0.109	0.118
e3	0.513	0.220	0.314

**Table 4:** Refinement of the three structures using reduced datasets *R* values from a refinement using conventional monochromatic data are included at the bottom of the table for comparison.

	Sanidine	Cs_2_[Pt(CN)_4_]H_2_O	ZSM-5
No. of reflections	1900	3093	9492
No. of unique reflections	675	1047	2615
Resolution range ()	0.7452.234	0.9162.344	0.9072.653
*R*_int_ †	0.053	0.097	0.075
*R*_sigma_ ‡	0.031	0.049	0.040
Completeness (%)§	80	84	80
No. of parameters	64	65	216
*R*1¶ [*F*_obs_ > 4(*F*_obs_)]	0.069	0.037	0.068
*R*1¶ (all)	0.074	0.037	0.157
*With monochromatic data:*			
*R*_int_ †	0.029	0.053	0.049
*R*1¶ [*F*_obs_ > 4(*F*_obs_)]	0.024	0.020	0.053
*R*1¶ (all)	0.026	0.020	0.057

† *R*_int_ = |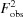 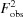
(mean)|/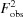.

‡ *R*_sigma_ = [(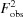)]/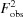.

§ Calculated for the resolution range 15.

¶ *R*1 = ||
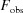
| |
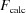
||/|
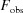
|.
